# Geo-epidemiology of malaria incidence in the Vhembe District to guide targeted elimination strategies, South-Africa, 2015–2018: a local resurgence

**DOI:** 10.1038/s41598-023-38147-0

**Published:** 2023-07-08

**Authors:** Sokhna Dieng, Temitope Christina Adebayo-Ojo, Taneshka Kruger, Megan Riddin, Helene Trehard, Serena Tumelero, Marc-Karim Bendiane, Christiaan de Jager, Sean Patrick, Riana Bornman, Jean Gaudart

**Affiliations:** 1 Aix Marseille Univ, IRD, INSERM, ISSPAM, SESSTIM, 13005 Marseille, France; 2 Swiss Tropical and Public Health Institute, Basel, Switzerland; 3 School of Health Systems and Public Health (SHSPH), University of Pretoria Institute for Sustainable Malaria Control (UP ISMC), University of Pretoria, Pretoria, South Africa; 4 Aix Marseille Univ, IRD, INSERM, ISSPAM, SESSTIM, APHM, Hop. La Timone, BioSTIC, Biostatistic & ICT, 13005 Marseille, France

**Keywords:** Malaria, Statistics

## Abstract

In South Africa, the population at risk of malaria is 10% (around six million inhabitants) and concern only three provinces of which Limpopo Province is the most affected, particularly in Vhembe District. As the elimination approaches, a finer scale analysis is needed to accelerate the results. Therefore, in the process of refining local malaria control and elimination strategies, the aim of this study was to identify and describe malaria incidence patterns at the locality scale in the Vhembe District, Limpopo Province, South Africa. The study area comprised 474 localities in Vhembe District for which smoothed malaria incidence curve were fitted with functional data method based on their weekly observed malaria incidence from July 2015 to June 2018. Then, hierarchical clustering algorithm was carried out considering different distances to classify the 474 smoothed malaria incidence curves. Thereafter, validity indices were used to determine the number of malaria incidence patterns. The cumulative malaria incidence of the study area was 4.1 cases/1000 person-years. Four distinct patterns of malaria incidence were identified: high, intermediate, low and very low with varying characteristics. Malaria incidence increased across transmission seasons and patterns. The localities in the two highest incidence patterns were mainly located around farms, and along the rivers. Some unusual malaria phenomena in Vhembe District were also highlighted as resurgence. Four distinct malaria incidence patterns were found in Vhembe District with varying characteristics. Findings show also unusual malaria phenomena in Vhembe District that hinder malaria elimination in South Africa. Assessing the factors associated with these unusual malaria phenome would be helpful on building innovative strategies that lead South Africa on malaria elimination.

## Introduction

In 2021, the World Health Organization (WHO) estimated 247 million malaria cases and 625,000 malaria deaths in 84 malaria-endemic countries. The WHO African Region represented about 95% of malaria cases and deaths^1^. South Africa is one of the countries in the WHO African Region^2^ affected by malaria and progressing towards elimination. The total population at risk of malaria in South Africa represents 10% or about six million inhabitants^3,4^ and is spread over only three provinces (out of a total of nine) endemic for malaria: Limpopo, Mpumalanga and KwaZulu-Natal^5–7^. In addition, malaria is seasonal, with a low transmission (winter) season between June and August and a high transmission (summer) season between September and May of the following year^5,8^.

From 2015 to 2019, South Africa recorded between 10,000 and 30,000 cases of malaria per year, and the national Department of Health plans to eliminate malaria (i.e. eliminate local transmission) by 2023^4^. However, there are challenges to achieving this goal^4^. Some researchers have differing views on the feasibility of malaria elimination, but generally agree that elimination in South Africa requires, among other things, good surveillance systems, the availability and the use of all effective intervention tools^9,10^. Furthermore, as countries, sub-national areas and communities approach the elimination phase of malaria, they are at different epidemiological stages, and therefore, intervention strategies need to be tailored to different regions within a country^11^. In addition, towards the elimination stage, transmission is generally low and spatially heterogeneous, thus requiring malaria analysis at the local, sub-district or village level^11,12^. Malaria transmission is heterogeneous across South Africa’s endemic provinces as showed in several studies^8,13,14^, and progress towards elimination has been similarly variable. Strategies should be tailored to the conditions of each province. Stratification of malaria intensity level is part of the WHO recommendations to accelerate the elimination process^11,15^. This stratification involves classifying geographical units according to local malaria transmission^11^. The aim of this approach is to group together geographical units that have not only similar intensity levels but also similar temporal dynamics.

Therefore, the functional approach^16,17^ based on the functional data method^18–20^, was adopted first for modelling the temporal evolution or dynamics of malaria incidence intensity, then, grouping them by similar intensity levels and temporal behaviour (dynamic) over time. The use of functional data method in public health and biomedical fields is discussed in the literature^21^. The principle of the functional data methodology is that observations (at a regular time lag or not) of a set of geographical units time series (country, health post, village, etc.) can be modelled by regularised smooth functions (called functional data or smooth curves) that are estimated with a single smoothing parameter determined from the sample. This allows the understanding of their underlying dynamics and allows the comparison of their trends to each other over time possible, because they will be analysed in the same scale^18^. Indeed, there was no automatic and objective way to compare thresholds and intensity levels between geographical units as shown in several epidemic studies^22–25^. In addition, this method is very useful in the context of the increasing use of sophisticated tools to collect and store large amounts of health data such as the DHIS2^26^. Thereafter, clustering algorithms^27^ can be applied on these functional data (malaria smoothed curves) to identify malaria incidence patterns according to the characteristics of their dynamics (local temporal trends). This would help to guide the development and implementation of targeted control strategies in the local context.

This functional approach consisting of the of the functional data method for modelling malaria evolution of geographical units and to apply clustering algorithm to them for identifying malaria patterns, was first developed to stratify malaria incidence level, and to identify malaria patterns at a village scale in Senegal^16^. Then, another study adapted this to stratify and identify malaria patterns at a village scale in Myanmar^17^. This suggests that this approach is applicable in the context of other endemic countries, such as South Africa.

In South Africa, the Limpopo province is the most affected^28^, particularly in the Vhembe District, where the malaria incidence in 2018 was 3.8 cases/1000 person-years^29^. In the literature, no studies have been conducted on malaria incidence at the locality level, the smallest geographical scale in South Africa, due in part to the difficulty of population availability at this scale^30^. As malaria elimination is approached, a finer scale analysis is needed to accelerate the results. Therefore, in the process of refining local malaria control and elimination strategies, the aim of this study was to identify and to describe the temporal dynamics of malaria incidence patterns at the locality level in the Vhembe District, Limpopo Province, South Africa.

## Methods

### Study area and dataset

**Figure 1:**
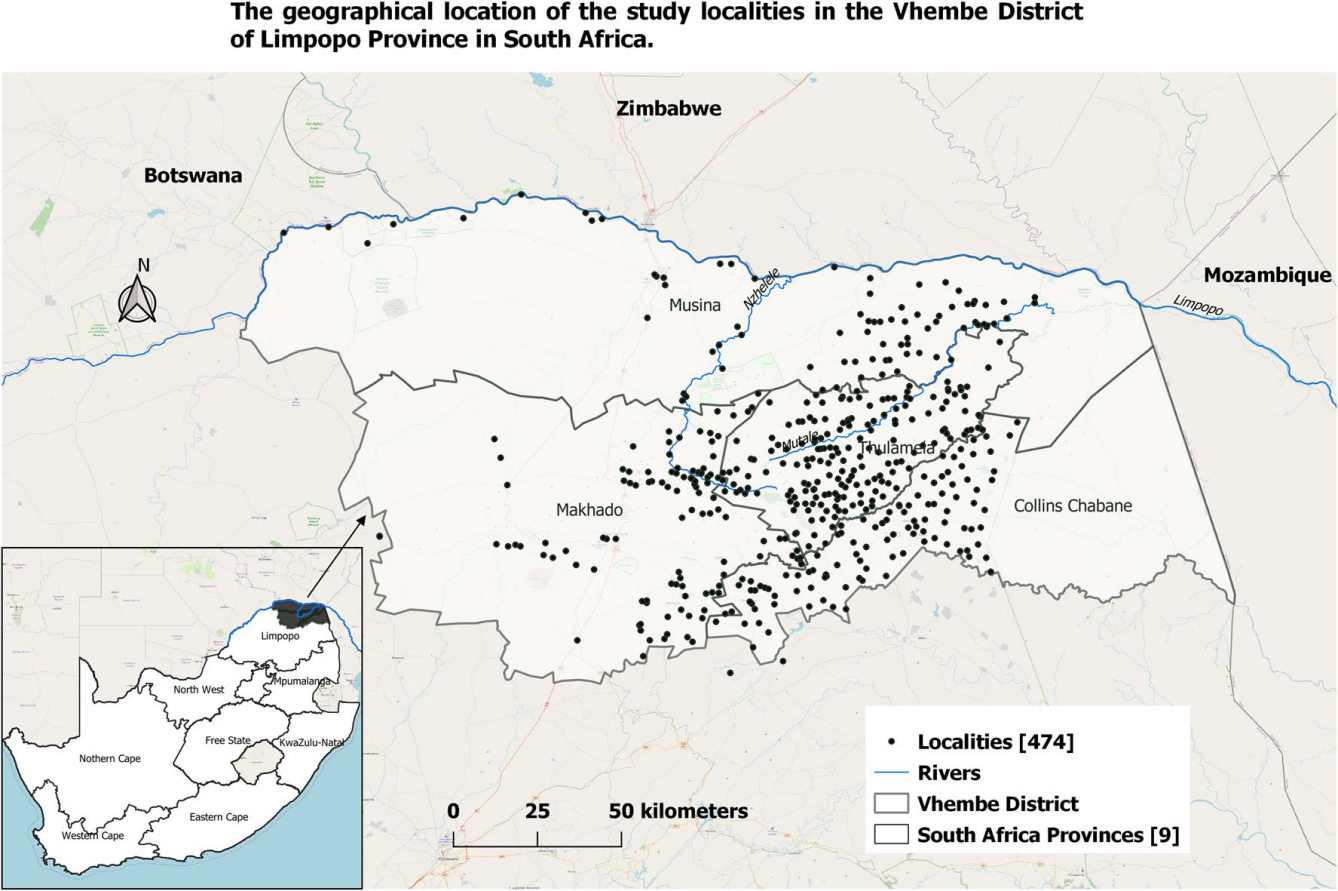
The study localities across Vhembe District in Limpopo Province, South Africa. Maps were produced by the authors using QGIS software (version 3.10.1., QGIS Development Team 2020. Open-Source Geospatial Foundation Project), OpenStreetMap and Shapefiles downloaded from websites^44,45^.

This study was conducted in 474 localities in the Vhembe District located in the Limpopo Province of northern South Africa (Fig. 1). These localities are scattered across the four local municipalities: Musina, Thulamela, Makhado and Collins Chabane with a total population of 1,294,722 from the last national census in 2011.

The period of this study was from July 2015 to June 2018 corresponding to three malaria seasons in South Africa: 2015/2016, 2016/2017 and 2017/2018. In this study it was essential to have for each locality, in addition to the malaria cases, the population to identify the incidence patterns and the geographical coordinates to locate them on the study site.

#### Malaria cases

Confirmed daily malaria cases were collected by the Limpopo Provincial Malaria Programme and stored in their information system^31^. A locality is present if at least one of its inhabitants has been diagnosed with malaria during the study period.

#### Population

To the best of our knowledge, all the studies done on malaria incidence dynamics in South Africa were at municipality, district or province scale, where official population data is fully available at these scales. However, this is not the case at the locality scale, which is the smallest geographical scale. The South African Department of Statistics^32^ had population data of some localities from the last census in 2011, but not for all localities. To complete them, others data sources^33^ and field investigations were used and combined with calculations based on known methods^32,34^ for all the years of the study period.

The starting point was the populations from the last national census in 2011. For each locality the information from the home municipality is provided. This was used to make a projection for each locality from 2015 to 2018 with the population growth rate of its belonging municipality corresponding to an average annual geometric variation rate^32,34^. This rate of change per municipality RateMun_ (2001–2011) was based on the last two national censuses of 2001 and 2011.

$$Pop_{i,year} = Pop2011_{i,year} * \left( {1 + {\text{RateMun}}_{2001 - 2011i} } \right)^{year - 2011}$$

#### Geographical coordinates

The missing geographical coordinates for some localities were obtained from the “geoview info” website^35^, and confirmed by field investigations.

### Statistical analysis

This analysis was based on an approach well described in^16,17^. Firstly, the weekly malaria incidence time series of each locality was determined over the study period. In order to have the main trend of their evolution with reducing variability, regularized smooth function (smooth curve or functional data) was fitted from each of them with the functional data method^18–20^.

Secondly, in order to identify patterns, hierarchical clustering algorithm with Ward’s method^36,37^ was performed on these 474 smooth functions through testing different measures of dissimilarity for having the most adaptive among them with the data. These measures of dissimilarity were the combination of temporal correlation and each of among Euclidean distance, Dynamic time warping and L2-metric in different levels of contribution^38–41^.

Lastly, for identifying the number of patterns, Principal Components Analysis (PCA)^42^ was performed on some validity indices (Dunn, Connectivity, Silhouette width and percentage of inertia R2)^41,43^ assessed over the clustering results done with each measure of dissimilarity by considering different potential number of patterns. The final number of patterns was the potential number of models from which a clustering result had obtained the best indices: great DUNN index, small Connectivity index, Silhouette width and percentage of inertia R2 close to 1.

Statistical analyses were performed using R software (version 4.1.3., R Core Team 2020. R Foundation for Statistical Computing, Vienna, Austria). Maps were produced by the authors using QGIS software (version 3.10.1., QGIS Development Team 2020. Open-Source Geospatial Foundation Project), OpenStreetMap and Shapefiles downloaded from websites^44,45^.

## Results

### Characteristics of Vhembe localities

The cumulative malaria incidence rate over the 474 localities in Vhembe was 4.1 cases/1000 person-years during the study period, from July 2015 to June 2018 (Table 1). At a localities scale, the median population was 1394 (IQR = [473; 2961]) and the median malaria incidence rate was 4 cases/1000 person-years (IQR = [1.4; 10.8]).

**Table 1:** Epidemiological characteristics of localities by seasons.

Characteristics^a^	Season 2015/2016	Season 2016/2017	Season 2017/2018	Study period
Cumulative incidence	1.0	4.0	7.4	4.1
Locality incidence	0.0 (0.0, 1.3)	3.3 (0.8, 9.4)	6.7 (2.0, 21.2)	4.0 (1.4, 10.8)

^a^Incidence in cases/1000 person-years; median and interquartile range.

Over the malaria transmission season, there was an increase in both cumulative and locality malaria incidences (Table 1).

### Main locality malaria incidence trends

**Figure 2:**
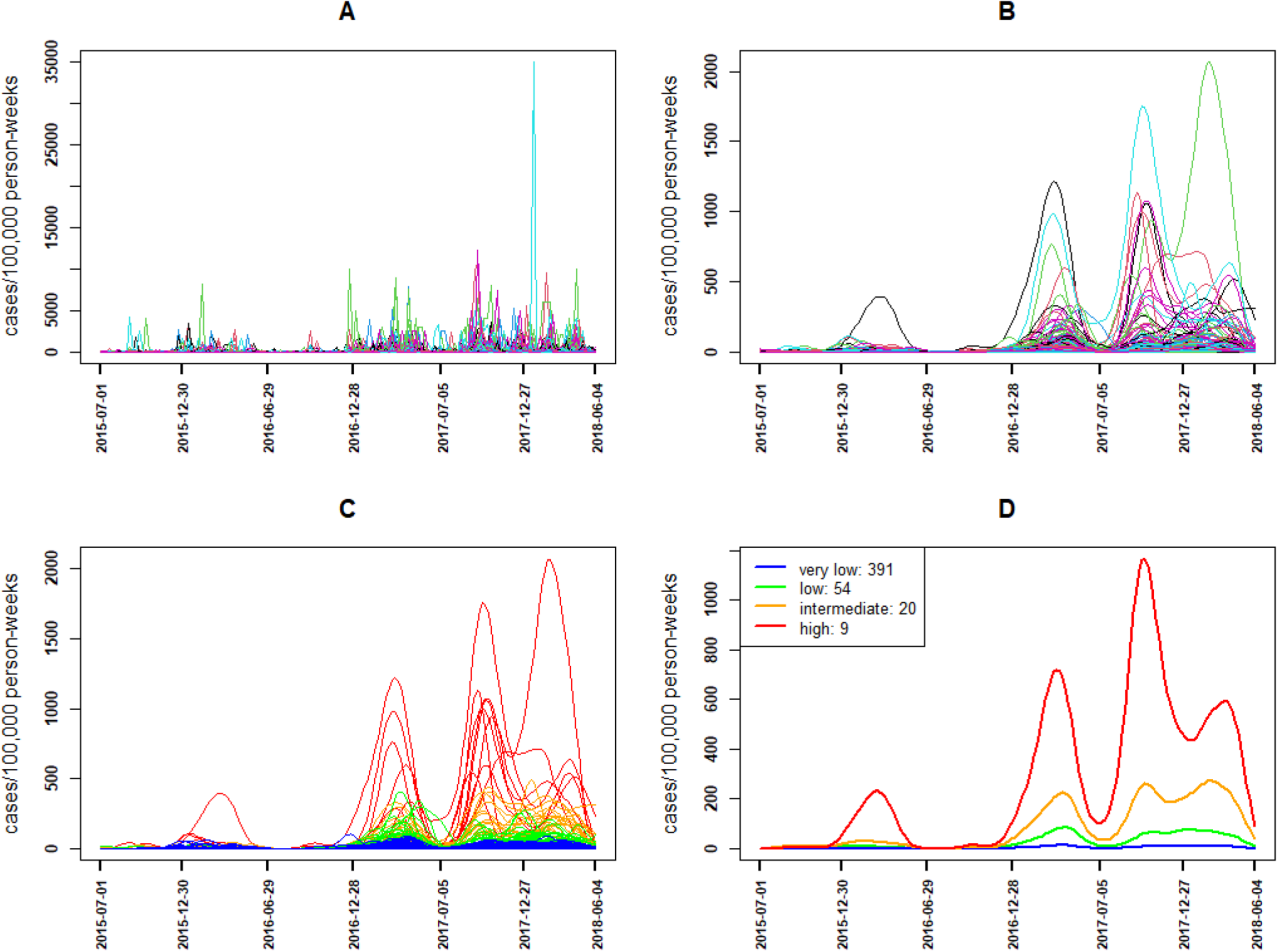
Smoothing and Identification of malaria incidence patterns from July 2015 to June 2018. Observed incidence localities (**A**), smoothed incidence localities (**B**), smoothed incidence localities of patterns (**C**) and smoothed cumulative incidence by pattern (**D**): very low (blue), low (green), Intermediate (orange) and high (red).

From July 2015 to June 2018, time series of weekly malaria incidence for each of the 474 localities was determined (Fig. 2, panel A). A substantial variability in malaria incidence was observed among localities, making it difficult to describe their main trends. To overcome this, a smoothing was carried out on these incidences with the method of functional data as described in the methodology section. All locality time series were smoothed on the same scale with K = 88 base functions and $\lambda$ = 144 as the smoothing parameter resulting in one smooth function (functional data or smooth curve) for each of the 474 localities in Vhembe District from July 2015 to June 2018 (Fig. 2, panel B). Thus, seasonal peaks were observed across high transmission period September to May with overall intensities increasing over time (Fig. 2, panel B). In addition, an unexpected increase was observed during the low transmission period June to August 2017.

### Identification and description of malaria incidence patterns

**Figure 3:**
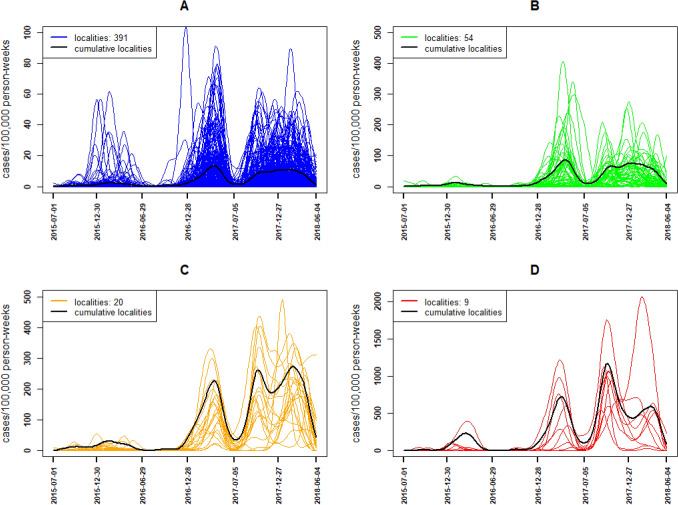
Smoothed incidence localities and cumulative incidence for each pattern from July 2015 to June 2018: Very low (**A**—blue), Low (**B**—green), Intermediate (**C**—orange) and High (**D**—red).

Malaria incidence patterns were obtained after performing the hierarchical clustering procedure described in the method section on these 474 smooth curves. Using dynamic time warping dissimilarity while considering mainly the temporal correlation in the hierarchical clustering algorithm and choosing four classes, gave a simultaneously better Dunn index, width Silhouette, Connectivity index and percentage of inertia regarding PCA results. Thus, four malaria incidence patterns were obtained: very low, low, intermediate and high pattern (Fig. 2, panel C and D;  3). It is observed that localities in the same pattern had the same temporal dynamic (Fig. 3).

**Figure 4:**
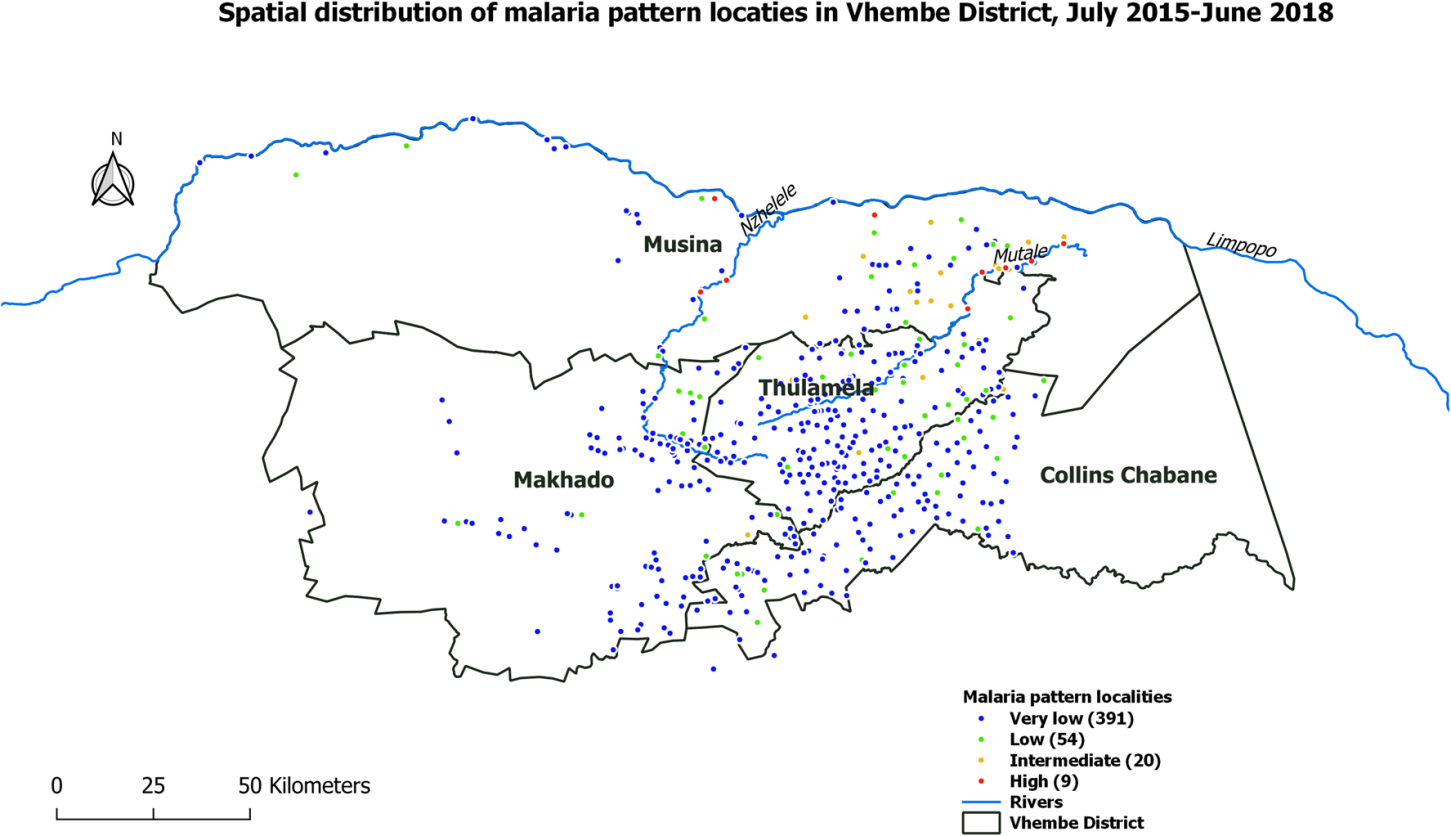
Spatial distribution of malaria incidence pattern localities in Vhembe District: very low (blue), low (green), intermediate (orange) and high (red). Maps were produced by the authors using QGIS software (version 3.10.1., QGIS Development Team 2020. Open-Source Geospatial Foundation Project) and Shapefiles downloaded from websites^44,45^.

The very low pattern included 391 localities, the majority of which were south of the Vhembe District, between the municipalities of Makhado and Thulamela (Fig. 4). Their incidences were the lowest in the study site (Figs. 2, 3). The cumulative malaria incidence observed in this pattern during these three seasons was 2.4 cases/1000 person-years (Table 2). The median incidence over the localities in this pattern was 3 cases/1000 person-years (1.1, 6.2) (Table 2). Their seasonal peaks were more important during the end of the second malaria season 2016/2017 but their epidemic period was wider during season 2017/2018.

**Table 2:** Characteristics of the four malaria incidence patterns.

Characteristic^a^	Very low, N = 391	Low, N = 54	Intermediate, N = 20	High, N = 9
Locality population	1602 (640, 3300)	674 (209, 1876)	467 (258, 1290)	120 (100, 338)
Cumulative incidence	2.4	16.2	54.1	181.2
Locality incidence	3.0 (1.1, 6.2)	21.7 (14.7, 33.3)	57.3 (40.6, 71.0)	211.3 (144.8, 237.2)
Cumulative incidence during season 2015/2016	0.63	3.12	10.63	58.34
Cumulative incidence during season 2016/2017	3	16	44	144
Cumulative incidence during season 2017/2018	4	29	108	337
Locality incidence during season 2015/2016	0.0 (0.0, 0.8)	0.5 (0.0, 3.4)	10.2 (5.0, 18.1)	33.5 (0.0, 110.5)
Locality incidence during season 2016/2017	2 (1, 6)	21 (11, 33)	41 (36, 51)	125 (60, 217)
Locality incidence during season 2017/2018	5 (1, 10)	39 (27, 54)	111 (78, 143)	317 (294, 507)

^a^Incidence in cases/1000 person-years; median (interquartile range).

The low pattern included 54 localities, the majority of which were between the north and south of the Vhembe District, in the municipality of Thulamela and Musina (Fig. 4). Their incidences were the lowest in the study site after the very low profile (Figs. 2, 3). The cumulative malaria incidence observed in this pattern during these three malaria seasons was 16.2 cases/1000 person-years (Table 2). The median locality incidence was 21.7 (14.7, 33.3) cases/1000 person-years (Table 2). Their seasonal peaks were also more important during the end of the second malaria season 2016/2017, but their epidemic period was wider during season 2017/2018.

The intermediate pattern comprised 20 localities, the majority of which were in the local municipality of Musina, very few of the localities in this pattern were present in the municipalities of Makhado and Thulamela (Fig. 4). Their incidences were the second highest after those observed in the high pattern (Figs. 2, 3). The cumulative malaria incidence observed in this pattern during these three seasons was 54.1 cases/1000 person-years (Table 2) while the median locality incidence was 57.3 (40.6, 71.0) cases/1000 person-years (Table 2). Their seasonal peaks were also important during the end of the third malaria season 2017/2018 and their epidemic period was wider during this season.

The high pattern included nine localities located north in the local municipality of Musina close to river systems (Fig. 4). The incidences in these localities were overall the highest during the study period (Figs. 2, 3). The cumulative malaria incidence observed in this pattern during these three seasons was 181.2 cases/1000 person-years (Table 2). The median locality incidence was 211.3 (144.8, 237.2) cases/1000 person-years (Table 2). Their seasonal peaks were more important during the beginning of the third malaria season 2017/2018 and their epidemic period were wider during this season.

Generally, very low and low patterns had the same temporal dynamics but at different levels of intensity, while intermediate and high patterns had slight differences at the end of the third season (Fig. 3). Furthermore, a general spatial trend was observed from south to north, with an increasing number of localities of the highest incidence patterns (from very low to high pattern) (Fig. 4).

### Local resurgence in malaria incidence patterns

From July 2017 onwards (Fig. 3) some characteristics appear to be more accentuated in the high and intermediate patterns, such as the presence of local rebound or recrudescence. These two patterns each have two recrudescence onset points (local minimum) around July and December 2017 during the last season. In addition, malaria incidence quantiles increased across patterns (from very low to high pattern) and transmission seasons (from season 2015/2016 to 2017/2018).

## Discussion

This work identified and described four different temporal malaria incidence patterns at the locality level in the Vhembe District during the three seasons; it further highlighted a local resurgence and an unexpected increase of malaria incidence during the study period.

In malaria endemic countries, as the elimination phase approaches, it is important to work on local spatial scales (villages and health posts, to name a few) and thin temporal scales (weekly)^46^. Working with these fine scales make it possible to reconstruct the reality of the epidemic beyond the administrative limits over time. In addition, this will make it possible to avoid missing certain malaria particularities that could not be analysed on larger spatial and temporal scales. This is due to the heterogeneity between localities belonging to the same region and their differences on temporal dynamics, which is not helpful for refining targeted control actions in space and time for achieving malaria elimination^47^.

Having data on these thin scales may be difficult in some countries or areas. However, in order to have relevant information for the development of control strategies, it would be necessary to conduct detailed analyses. This requires the implementation and effective monitoring of a data collection system^12,48^. According to the WHO, there is no single intervention or set of interventions that will achieve malaria elimination in all countries. Rather, an arsenal of interventions tailored to the intensity and dynamics of malaria transmission in each country must be identified and used to achieve and sustain elimination. The effectiveness of interventions varies depending on where and when they are applied. In addition, accurate stratification of malaria intensity in local context is essential for effective targeting of interventions.

Therefore, the introduction of the functional approach in this framework of epidemiology may be an alternative. Indeed this approach answers simultaneously to a set of technical limits of classical methods^49,50^ as mainly the possibility to model simultaneously a large dataset with high dimension, the possibility to compare the spatio-temporal dynamic of malaria between them while analysing them in the same scale, the possibility to handle missing data or to deal with irregular observation times by working with estimated malaria incidence in real time during the study period, the possibility to appropriately classify malaria time series for clustering geographical units with the same intensities and same temporal dynamics^16,17,51,52^. Indeed, it is important to capture how malaria incidence evolves over time across localities. Even if two localities have the same level of malaria incidence intensity, they would need different actions due to how they evolve over time. Moreover, the mathematical properties underlying this functional method also allow the analysis of malaria speed and acceleration corresponding to the first and second derivatives of the incidence curves. These derivatives make it possible to determine epidemiological indicators such as the dates of onset, acceleration, peak, slowdown and end of the epidemic^16,53,54^. Although the latter points are not investigated in this study, it was important to show the potential of this method for further analysis.

One of the limitations of this approach, for operational aspects, could be the dispersion of the localities of the same pattern over the geographical space. However, this limitation can be overcome with good local coordination, i.e., at district or communal level, intervention strategies deployed on local targets and well organised priorities^46^. Depending on the epidemiological realities of the countries, some characteristics would be different like the number of patterns, the temporal and spatial dynamic of patterns and epidemiological indicators^16,17^.

Malaria transmission in South Africa is seasonal, with the low transmission period (winter) starting in June and ending in August; and the high transmission period (summer) starting in September and ending in May of the following year^5^. Generally, malaria cases start to increase in October, peak in January and February, and then decrease around May^31^. Our results showed that the last period of low transmission (June–August 2017), which was expected to have a very low level of incidence, had a sharp increase especially for the intermediate and high patterns and an unexpected peak around October 2017 in addition to the seasonal peak around February 2018: resurgence occurrence. It would be enlightening in this context to understand the factors that would be associated with the high intensity of incidence during the low transmission period of the 2017/2018 season and the occurrence of the resurgent series. These results open perspectives on the search for potential factors associated with these phenomena. However, several studies^55,56^ in South Africa has linked the recent malaria resurgence to climatic variables such as rainfall and temperature, to the mosquito abundance and arrival of new mosquito species. Under suitable conditions, mosquitoes bite more aggressively to produce more eggs, and in the process, transmit malaria faster than usual.

Furthermore, the spatial distribution of localities in Vhembe District showed that the further north one goes towards the Limpopo River (Mutale), the more one encounters malaria patterns with a high level of incidence indicating a spatial trend in malaria incidence. Specifically, the high pattern localities which are along the Mutale, Limpopo and Nzhelele Rivers (Fig. 4). One of them, Gwakwani, which was an unrecognised hotspot, is currently benefiting from a research project to better understand the local risk factors^57^. It has also been established that in South Africa low-lying areas (around 1000 m) are more affected by malaria^5,58^, as is the case in the Limpopo River valley and its tributaries. In addition, this northern area borders on the malaria-endemic Zimbabwe and Mozambique^59^, and imported cases are frequently reported due to economic migration during the rainy season (agriculture)^29^. In terms of malaria control, South Africa has continued to apply indoor residual spraying as their primary means of control in the country^10^.

Studies have shown the relationship between climatic, environmental, entomological, preventive and social factors on malaria incidence in South Africa^6,8,14^. The use of these data in this work could help to identify the factors associated with the locality malaria patterns and the dynamic characteristics of the factors associated with temporal increases and decreases (variations) in incidence in these localities. This latter point can be investigated by using functional regression^18–20,40^ that allows finer results on the effects of factors on the dynamic of malaria.

This study also proposed methodological tools for guiding intervention strategies by targeting priority areas and relevant dates for implementation and highlighted the need of:

- Improving the data collected at local scale across health information systems,
- Improving the availability of data population at local scale by official sources,
- Improving local environmental, economic and social surveillance for better investigating their effect at local scale,
- Additionally, defining and adapting health policies at local scale by:
  - Targeting priority areas using malaria patterns,
  - Identifying their dynamic characteristics and the factor effects over time,
- Implementing strategies according to their characteristics and identifying the appropriate times for each malaria pattern to apply the targeted control actions (allowed by the regularised smooth functions that estimated the incidence in real time even where they were not observed).

## Conclusion

This study showed four temporal dynamics of malaria in Vhembe District with different characteristics. It also highlighted unusual malaria phenomena in Vhembe District such as a high level of incidence during a low transmission season and the occurrence of a series of resurgence. South Africa has reached the malaria pre-elimination stage, based on the development of access to diagnostics, treatment and vector control. However, the recent resurgence of malaria in remote areas hinders malaria elimination, highlighting the need for assessing the resurgence determinants and developing innovative targeted malaria elimination strategies in keeping with the local and global goals on eliminating malaria.

## Data Availability

The datasets analysed during the current study are available from the corresponding author on reasonable request.

## Abbreviation

IQR: Interquartile range
